# HRU-Net: A Transfer Learning Method for Carotid Artery Plaque Segmentation in Ultrasound Images

**DOI:** 10.3390/diagnostics12112852

**Published:** 2022-11-17

**Authors:** Yanchao Yuan, Cancheng Li, Ke Zhang, Yang Hua, Jicong Zhang

**Affiliations:** 1 School of Biological Science and Medical Engineering, Beihang University, Beijing 100191, China; 2 National Engineering Research Center of Telemedicine and Telehealth, Xuanwu Hospital, Capital Medical University, Beijing 100053, China; 3 Hefei Innovation Research Institute, Beihang University, Hefei 230012, China; 4 Department of Vascular Ultrasonography, Xuanwu Hospital, Capital Medical University, Beijing 100053, China; 5 Beijing Diagnostic Center of Vascular Ultrasound, Beijing 100053, China; 6 Center of Vascular Ultrasonography, Beijing Institute of Brain Disorders, Collaborative Innovation Center for Brain Disorders, Capital Medical University, Beijing 100069, China

**Keywords:** carotid ultrasound, plaques segmentation, CNN, atrous convolutions, transfer learning

## Abstract

Carotid artery stenotic plaque segmentation in ultrasound images is a crucial means for the analysis of plaque components and vulnerability. However, segmentation of severe stenotic plaques remains a challenging task because of the heterogeneities of inter-plaques and intra-plaques, and obscure boundaries of plaques. In this paper, we propose an automated HRU-Net transfer learning method for segmenting carotid plaques, using the limited images. The HRU-Net is based on the U-Net encoder–decoder paradigm, and cross-domain knowledge is transferred for plaque segmentation by fine-tuning the pretrained ResNet-50. Moreover, a cropped-blood-vessel image augmentation is customized for the plaque position constraint during training only. Moreover, hybrid atrous convolutions (HACs) are designed to derive diverse long-range dependences for refined plaque segmentation that are used on high-level semantic layers to exploit the implicit discrimination features. The experiments are performed on 115 images; Firstly, the 10-fold cross-validation, using 40 images with severe stenosis plaques, shows that the proposed method outperforms some of the state-of-the-art CNN-based methods on Dice, IoU, Acc, and modified Hausdorff distance (MHD) metrics; the improvements on metrics of Dice and MHD are statistically significant (*p* < 0.05). Furthermore, our HRU-Net transfer learning method shows fine generalization performance on 75 new images with varying degrees of plaque stenosis, and it may be used as an alternative for automatic noisy plaque segmentation in carotid ultrasound images clinically.

## 1. Introduction

Atherosclerosis is a progressive disease associated with the deterioration of carotid arteries, which is caused by hypertension, diabetes, obesity, etc. In particular, a clinical feature of this disease is the formation of plaques [1,2] in the intimal layer of the carotid artery walls. It is noteworthy that plaques can decrease blood flow and may break, inducing strokes [3,4]. Intima-media thickness (IMT) is an early crucial stage of plaque formation, so carotid IMT is also used to evaluate the change of the carotid artery [5]. However, IMT is only useful in the early stage of atherosclerosis; it does not predict cardiovascular risk [6], and it also cannot assess the changes in morphology and echogenicity of carotid plaques, whereas the stenosis degree of the internal carotid artery is a reliable plaque measurement pattern for assessing stroke risk [7] and then deciding whether carotid endarterectomy is required [8].

Although it is recognized that the degree of stenosis should be measured, more attention should be paid to vulnerable or unstable carotid plaques, as they are determinant factors for stroke risk. Concretely, an unstable plaque may induce local thrombosis or distal embolization because of the debris of a ruptured plaque [9]. Because of the low-cost and nonradiative and noninvasive features, B-mode ultrasound technique has been widely used for the diagnosis of atherosclerosis [10,11] in the carotid artery. In particular, sonographers capture the longitudinal view of carotid artery, and then the length and thickness of plaques are measured manually; moreover, the echo characteristics of plaques can be visually evaluated for further analysis. 

**Figure 1:**
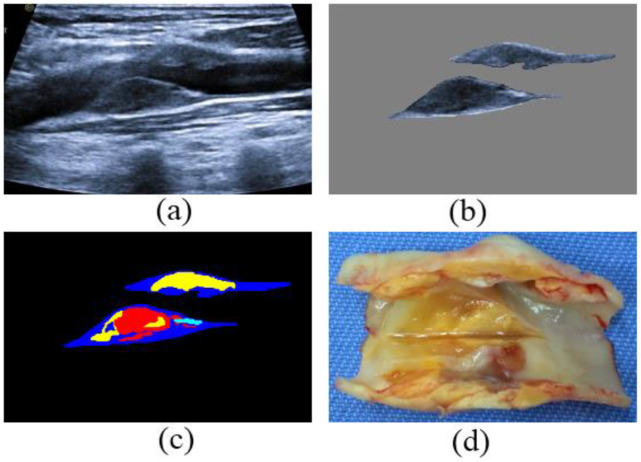
Carotid vulnerable plaques from a patient: (**a**) 2D ultrasound images before endarterectomy, (**b**) the solo ultrasound plaques, (**c**) the labels (blue, light blue, yellow, and red denote fiber, calcification, lipid, and hemorrhage components in the plaques, respectively) of ultrasound plaque components in (**b**), and (**d**) the plaques specimen after endarterectomy of the patient.

For instance, vulnerable plaques with severe stenosis from one patient are shown in four forms in Figure 1. Specifically, as shown in Figure 1a,b, the plaques almost plug the lumen on the near and far walls of the artery in the ultrasound image. The fibrous cap of the plaques becomes thin due to the richness of lipids nuclei and hemorrhage components, which are clearly depicted in Figure 1c. After the endarterectomy of the patient, the plaque specimen consists of complex pathological components, as shown in Figure 1d. Noteworthy, the label compositions of the plaques in Figure 1c were determined by the sonographers according to their clinical experience and the plaque specimen in Figure 1d.

It is noteworthy that plaque segmentation helps separate the diagnostic region of interest (ROI) from the background, and texture features (such as low echo, high echo, echoless, and isoechoic or mixed echo) of the plaque are subsequently quantitatively measured. However, manual plaque segmentation is laborious. Additionally, the segmentation accuracy heavily depends on the experience of the clinicians, and it may suffer from inter- and intra-rater variabilities [12], resulting in a limited reproducibility. Therefore, many studies have been devoted to developing semi-automated [13,14] and automated [15,16] plaques or intima-media complex (IMC) segmentation algorithms to solve this dilemma.

**Figure 2:**
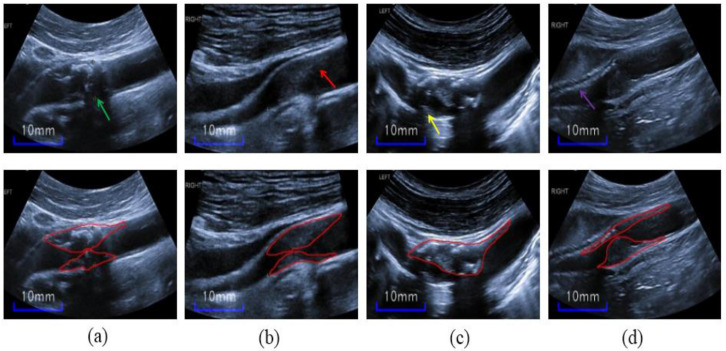
Carotid plaques in ultrasound images from four patients. Top row: (**a**) The green arrow indicates acoustic shadow, (**b**) the red arrow indicates speckle noise, (**c**) the yellow arrow indicates artifacts, and (**d**) the purple arrow indicates the ambiguous boundary between the plaques and IMC. Bottom row: The red outlines superimposed on the images denote the plaques contours.

However, severe stenosis plaque segmentation is quite challenging, and the difficulties are summarized threefold. (1) Heterogeneities of inter- and intra-plaques: Specifically, the inter-subject variations of plaques can be large; they have no common in shape and echo characteristics among different individuals shown in Figure 2. Moreover, the mixed echo (as shown in Figure 2c) of the plaques from one subject makes the shapes of plaques more indiscernible. (2) Obscure boundaries of plaques: In ultrasound images, carotid plaques are contaminated by low echo plaque region, speckle noise, artifacts, and acoustic shadow during image acquisition. (3) The ambiguous boundary between the plaque and intima-media thickening region increases the difficulty for plaque segmentation; moreover, some artifacts may be mistaken for plaques because of the similarity between plaques and artifacts.

To achieve ideal segmentation performance, traditional approaches require complicated steps, which may be recalibrated for new images. Nowadays, convolutional neural network (CNN) based deep learning methods [17,18,19] are becoming increasingly more advantageous for semantic segmentation tasks; however, these methods need numerous labeled images for training. Specially, CNN-based plaque or IMC segmentation methods [20,21,22] obtain satisfactory results, but these methods need manual ROI preprocessing, and they require more ultrasound images for training. Moreover, our plaque segmentation task has no anatomy prior compared to IMC segmentation [23,24,25], which increases the difficulty for plaque segmentation. Moreover, the limited images used in our study may increase the difficulty for the common CNN-based methods.

To overcome these issues, in this study, we first manually cropped the blood vessel in carotid ultrasound images, as the plaques are only in the inner of the artery; thus, the cropped images are provided as a data augmentation regularization method for the limited noisy images. Secondly, transfer learning [25,26,27] can eliminate training from scratch with a large labeled dataset, which is suitable for the limited labeled images. Thus, we use the ResNet-50 [28] as our base encoder network for fine-tuning pretrained parameters, exploiting the cross-domain knowledge. Moreover, ResNet-50 was trained on millions of natural images because it has rich and inter-class analogous textures, and it could serve as an alternative for our small ultrasound data. 

Thirdly, artifacts and speckle noise have semblable appearances with plaques, and dilated convolutions [29,30,31] (or atrous convolution) can enlarge the fields without introducing additional parameters. Different from the combinations of large dilated rates in References [32,33], we propose small hybrid atrous convolutions, referred to as an HACs module, and the dilated convolutions with various small rates can acquire various receptive fields for high-level semantic contexts and unearth implicit discrimination information. 

Our contributions are as follows:

(A) We utilized cropped carotid blood vessel (CBV) images as a data augmentation mode, and CBV provided plaque position constraint during data training; moreover, the transfer learning from trained ResNet-50 was made use of for ultrasound plaque segmentation.
(B) Hybrid atrous convolutions (HACs) were used on the last three high-level layers of the ResNet-50, and the HACs could obtain more receptive fields to discriminate similar textures between plaques and speckle noise.
(C) We tested our model on noisy ultrasound images with varying degrees of carotid stenosis, showing a fine segmentation performance.

## 2. Related Work

### CNN-Based Segmentation for Carotid Ultrasound Images

The CNN-based deep-learning method achieves significant success in image segmentation on account of its automatic feature-extraction ability. Specifically, U-Net [19] is a popular baseline method for semantic segmentation, and U-Net-based methods have been used for lumen–intima boundary (LIB) and media–adventitia boundary (MAB) segmentation [23,34] in carotid ultrasound images. Additionally, Zhou et al. [24] combined a voxel-FCN and a continuous max-flow postprocessing algorithm to segment the MAB and LIB from 3D ultrasound images. More recently, we proposed CSM-Net [35] to segment the intima–media complex and lumen with attention mechanisms and an improved loss function in 2D ultrasound images. However, the LIB and MAB biomarkers cannot sufficiently denote the plaque progress.

Vila et al. [22] proposed a single-step DenseNet to segment plaques and estimate the carotid IMT; however, the IMT measurement method could overestimate the values of oblique CA. Recently, A UNet++ ensemble algorithm [20] was proposed to segment plaques from small data and test on another dataset. Furthermore, two U-Net models [36] were trained by using two different ground-truth datasets for plaque segmentation [21] focused on plaque segmentation on the far wall of the internal carotid artery (ICA), using solo deep learning (SDL) and hybrid deep learning (HDL) models; moreover, they used U-Net to demonstrate that “Unseen AI” was in close proximity to “Seen AI” [37]. In brief, these methods need manually preprocessing for ROI, and the segmentation task has morphological consistency, which reduces the segmentation difficulty.

## 3. Methods

**Figure 3:**
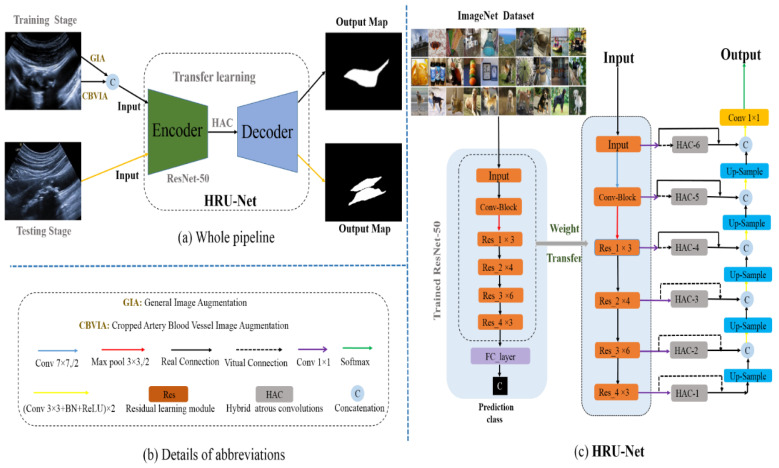
HRU-Net transfer learning for segmenting carotid artery plaques in ultrasound images.

The proposed method consists of a ResNet-50 transfer-learning encoder, U-Net-based [19] HAC decoder, and data augmentation, as shown in Figure 3. During the training stage, the framework takes the general image augmentation (GIA) and the cropped-artery-blood-vessel image augmentation (CBVIA) as input. 

The pretrained ResNet-50 network serves as an encoder for transfer learning, and the decoder is a skip-connection style like U-Net [19]. The last three deep-encoder layers, consisting of high-level semantic information, actually pass the HACs (hybrid atrous convolutions) modules (Figure 3) to receive more receptive fields and suppress irrelevant features. During the testing stage, we only input the original images without any image augmentation in the trained network to obtain output results.

### 3.1. Image Augmentation

#### 3.1.1. General Image Augmentation (GIA)

The standard CNN has the feature of translation invariance; however, the individual CNN has no rotation invariance in order to avoid overfitting and improve the generalization of deep neural networks; using limited images, we performed the following general data augmentation operations on the original image (I), namely flip horizontal (H) and flip vertical (V), +30° ($R_{+30}$) and −30° ($R_{-30}$) rotations, and 180° ($R_{180}$) rotation. 

These image-transforming operations can not only enhance the dataset size for the network training but also provide various view angles of carotid ultrasound images. Moreover, the corresponding labels are performed by the same transforming operations. Herein, N is the number of the raw images, and then the augmented images increase to 6*N images. It is formally expressed as follows:

(1) $$\mathrm{GIA}=H\left(I\right)+V\left(I\right){+R}_{180}\left(I\right){+R}_{+30}\left(I\right){+R}_{-30}\left(I\right)+I$$

#### 3.1.2. Cropped-Blood-Vessel Image Augmentation (CBVIA)

**Figure 4:**
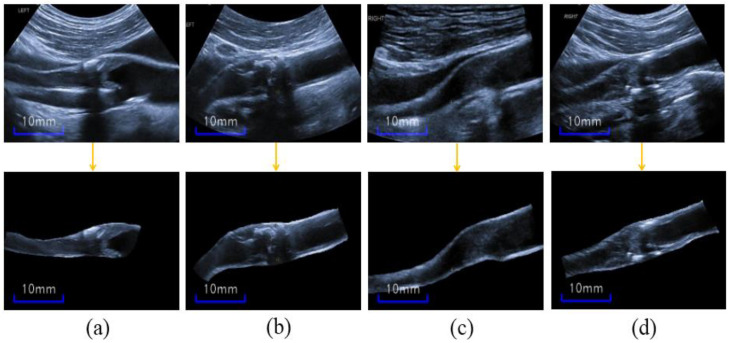
(**a**–**d**) Original ultrasound images (the first row) and the corresponding cropped-artery-blood-vessel images (the second row).

The carotid ultrasound images in our study are limited for training, and the segmentation network may be overfitted, despite the general image augmentation. Furthermore, our complex ultrasound images, which have different types of artifacts and inhomogeneous plaque appearances, make it impossible to mimic the mask artifacts used in Reference [34]. As shown in the first row of Figure 4, the plaques are not well distinguished given the blurry blood vessels and unclear plaque boundaries; thus, the areas outside the artery blood vessel may be misclassified as plaques.

Because plaques are only in the inners of arteries, this feature can be used as a prior knowledge for plaque segmentation. To take full advantage of this crucial information, we cropped the blood vessel (CBV) manually from original ultrasound images, as shown in the second row of Figure 4. Thus, the CBV images are provided as a data augmentation during only the training stage. In addition, only the original image without any augmentation was inputted into the network in testing. Noteworthy, the cropping operation is actually the manual segmentation of the blood vessel for image augmentation.

Moreover, the CBV images’ augmentation (CBVIA) provides the direct contrast information of blood-vessel boundary, and the segmentation network may concentrate on the inner of the artery during the training stage. Moreover, CBVIA also directly points out the actual boundary around the shadow area. Finally, the formula of CBVIA is expressed as follows:

(2) CBVIA=V(CV(I))+CV(I)

where $\mathrm{CV}\left(I\right)$ represents the operation of cropping blood vessel of the carotid ultrasound image, V(.) denotes the flip vertical transformation, and the number of CBV images is 2 × N. 

### 3.2. ResNet-50 Based U-Net (RU-Net) Transfer Learning

**Figure 5:**
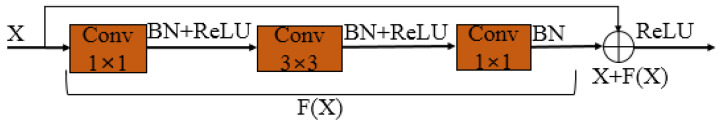
Residual learning module (Res). Conv, BN, and ReLU denote convolution, batch normalization, and rectified linear unit, respectively.

ResNet-50 [28] was introduced to address the gradient vanish and exploding problem, and it was trained on the ImageNet [38] dataset that consists of 1000 object classes; the trained network has the capacity of identifying fine-grained similar textures of natural images. Figure 5 shows the residual learning module, called Res, which is defined as follows:

(3) $$y=F\left(x,\;W\right)+x$$

where x and y are the input and output maps of corresponding layers; function F (x, W) represents the residual mapping to be learned, as shown in Figure 5; and W is the parameter. In Figure 3c, the encoders from ResNet-50 include 4 residual blocks Res_1 to Res_4. Noteworthy, the number of maps increases largely along with the increase of the residual learning block.

Deep learning from scratch may be tedious; moreover, limited datasets could lead to overfitting and a poor test result. It is noteworthy that fine-tuning [25] on a trained network obtains outstanding results despite the size of training sets. In other words, the source domain (pretrained natural images) can be transferred to the target domain (medical image tasks) by fine-tuning the trained parameters.

Inspired by this, we adopted the pretrained ResNet-50 as an encoder transfer learning, as shown in the left part of Figure 3c. Specifically, the discrimination ability of analogous textures of ResNet-50 is exploited as prior knowledge. To decrease the parameters and speed up the training time, we appl a 1 × 1 convolution on the output of each residual block, and the reduced channel numbers are listed in Table 1. Subsequently, each block is processed with skip concatenation in the decoder, like in the U-Net network, as shown in Figure 3c. Therefore, we named the base encoder–decoder structure RU-Net. 

### 3.3. Hybrid Atrous Convolutions (HAC) Module

A convolutions neural network can extract features in the images with its characteristics of local awareness and shared weights. However, this feature may face a dilemma in the deep semantic layer due to the small fixed-size receptive field of the convolutions kernel. In other words, the common convolution operation cannot obtain fine performance for the higher-layer semantic information. It is worth noting that dilated convolutions [30,31] can expand receptive fields (RFs) by inserting zeros in standard convolutions without introducing additional parameters. For a random pixel, p (m, n), in a feature map, the convolution between the p (m, n) and a kernel, h, is defined as follows:

(4) $$p(m,n)*h=\sum_{i=1}^{K}\sum_{j=1}^{K}p\left(m-\frac{r\left(K+1\right)}{2}+\mathrm{ri},\;n-\frac{r\left(K+1\right)}{2}+\mathrm{rj}\right)h\left(i,j\right)$$

**Figure 6:**
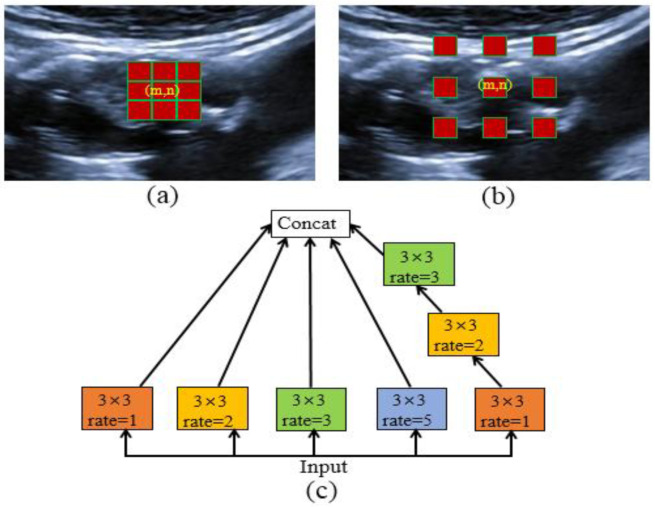
Schematic diagram of atrous convolutions. A pixel point $\left(m,n\right)$ in a convolution with kernel 3 × 3, atrous rate r = 1 (**a**) or r = 2 (**b**). (**c**) Hybrid atrous convolutions (HACs) module with convolution 3 × 3; atrous rate 1, 2, 3, and 5; and a cascaded atrous rate 1, 2, and 3.

The kernel, h, is a K×K (generally, K is odd) trainable matrix, and r is the atrous rate. When r = 1, Equation (4) is a standard convolution. Further, the position $\left(m,n\right)$ is the center of the convolution kernel, and a larger r indicates a greater receptive field of the convolution. For example, when K = 3, r = 1, or 2, the red squares shown in Figure 6a,b are the convolution kernels superimposed on a carotid ultrasound image, and the latter has a larger receptive field with the same parameters. However, a gridding issue exists in the dilated convolutions because of inserting zeros between each pixel in the convolutional kernel. 

To address this problem, HDC [29] was proposed by applying 3 × 3 convolutions with sequential dilated rates of 1, 2, and 3 to obtain an RF of 13 × 13. It outperforms the big atrous rates’ ASPP [32] and big dilated rates used in Reference [30]. Moreover, we suppose that small individual rates such as 1, 2, 3, and 5 obtain more differentiable semantic features about plaques and noises on the deep semantic layers, such as Res_2 to Res_4. Therefore, we integrated these atrous convolutions, called hybrid atrous convolutions (HACs), as shown in Figure 6c. 

Firstly, we used the HAC module on the last three high-level encoding layers, as shown in Figure 3, rather than only the highest layer in Reference [33]. In particular, the combinations of small dilation factors help search for the local and global structures of inhomogeneous plaques, especially for the Res_4 blocks. Moreover, the HACs could better obtain the long-range dependency of features maps on Res_4 blocks compared to tedious Non-Local Net [39]. 

Furthermore, the high semantic layers Res_2 and Res_3 blocks further obtain more RF by using HAC modules for inter-class differentiation around the blurry plaque boundaries. In summary, these small dilated convolutions improve the performance of plaque segmentation in ultrasound images. Thus, the three HACs embedded in the RU-Net are shown in the middle part of Figure 3c, and the network is finally named HRU-Net.

### 3.4. Dataset

The dataset used in this study was retrospectively obtained from the Vessel Ultrasound Diagnostic Department of Xuanwu Hospital, Capital Medical University. A total of 115 images (90 subjects, with a mean age of over 60, years including men and women) were enrolled in our experiments; these images were diagnosed with mild-to-severe (10–99%) carotid stenosis. Concretely, there were 30 images with mild-to-moderate stenotic plaques from 30 subjects and 85 images from 60 subjects with moderate-to-serve stenotic plaques.

The subjects with vulnerable plaques underwent carotid endarterectomy (CEA), following the protocol previously reported [40], and the vulnerable plaques were diagnosed according to the criteria described by Naghavi et al. [41]. Moreover, the carotid artery ultrasonography performed on these subjects was by experienced ultrasound physicians (with more than 10-year of experience). In particular, both the common carotid artery (CCA) (including the bifurcation) and the internal carotid artery (ICA) were imaged in our study to obtain intact plaques.

The original data were 2D longitudinal ultrasound type with a resolution of 1024 × 768, acquired using HITACHI ultrasound systems (Ascendus, HITACHI Inc., Tokyo, Japan) with a 4.0–8.0-MHz micro-curvilinear array probe (EUP-C734) and a 3∼12 MHz ultra-wideband linear array probe. Specially, the curvilinear array probe is provided for the fat patients or the patients with stubby neck, as the curvilinear array probe can enhance the detection depth and capture a wider capture angle for these patients compared to the linear array probe, and the choice of the probe depends on the type of the patient’s figure. 

We cropped the images to remove the personal identifiers and resized the unified size of 512 × 320. The plaque labeling was performed by two experienced doctors (with more than 10-years of experience), using the ITK-SNAP [42] software. To be specific, the images were mainly labeled by an ultrasound technician with about 10 years of experience and then examined and corrected by a senior ultrasound technician with more than 10 years of experience. Thus, this can reduce the false labeling and make the label more reliable.

Two types of labels were used in our experiment: firstly, the plaques’ contours were drawn manually as closed curves; secondly, adventitia contours of the internal or common carotid artery were delineated manually as closed curves. Furthermore, we extracted and filled the two types of contours by the algorithms in Open-CV library, and then the plaque labels and cropped artery blood vessel (CBV) masks were completed; the CBV masks were multiplied by the original images to obtain the CBV images. 

### 3.5. Metric

The metrics used in this study for assessing the segmentation results of the plaques were the Dice Coefficient (Dice) (Equation (5)), Intersection Over Union (IoU) areas (Equation (6)), accuracy (Acc) (Equation (7)), and modified Hausdorff distance (MHD) [43] (Equation (8)).

(5) $$\mathrm{Dice}=\frac{2|L\cap S|}{\left|L|+|S\right|}$$

(6) $$\mathrm{IoU}=\frac{\left|L\cap S\right|}{\left|L|+|S|-|L\cap S\right|}$$

where L and S denote the ground truth and the segmentation result, respectively. The Dice and IoU values are between 0 and 1:

(7) $$\mathrm{Acc}=\frac{\mathrm{TP}+\mathrm{TN}}{\mathrm{TP}+\mathrm{TN}+\mathrm{FP}+\mathrm{FN}}$$

where TP, TN, FP, and FN are the true positive, true negative, false positive, and false negative, respectively. The Acc value is between 0 and 1:

(8) $$\mathrm{MHD}=\max\left\{d\left(L_{b},S_{b}\right),d\left(S_{b},L_{b}\right)\right\}$$

where

(9) $$d\left(L_{b},S_{b}\right)=\frac{\sum_{a\in L_{b}}d\left(a,S_{b}\right)}{N_{a}}$$

The MHD outperforms the Hausdorff distance (HD) for distance evaluation between the boundaries of two objects; the lower value shows the better result. In Equations (8) and (9), $L_{b}$ and $S_{b}$ denote the boundaries of L and S. Specifically, for the definition of d ($L_{b}{,\;S}_{b}$), the $d(a,S_{b}$) denotes the minimum value of Euclidean distance between the point a on the boundary $L_{b}$ and all points on the boundary $S_{b}$, and $N_{a}$ is the number of points on the boundary $L_{b}$. The definition of d ($S_{b}{,\;L}_{b}$) is the same as d ($L_{b}{,\;S}_{b}$). 

### 3.6. Statistical Analysis

The MATLAB 2017 was used to perform paired *t*-tests in order to validate whether there is a statistical difference between two methods, and we used Bonferroni correction to correct for type 1 errors for multiple *t*-tests. Furthermore, we use total plaque-area error (ΔTPA) to analyze the segmentation performance.

### 3.7. Implementation Details

We utilized a Tesla v100 GPU for data training, using the Keras framework and TensorFlow as the backbone. We randomly chose 40 ultrasound images (from 40 different patients) with severe carotid stenosis for training, and a 10-fold cross-validation was adopted. In particular, 4 out of 40 images were for testing, while others were for training, and the result was the average of the results of 40 images. In summary, we obtained 320 images after the GIA (240) and CBVIA (80). Specifically, in the implementation of cross-validation, we used 288 images for training and the other 4 original images for testing. Furthermore, the remaining 75 images from 50 subjects are used for a further test of the 10-fold models.

The one-hot code was adopted for pixel softmax classification. The Adam optimizer was chosen for optimization with the β1 = 0.9, β2 = 0.999, ε = 1 × 10^−8^. Besides, the learning was set to 0.0001 for fine-turning, and the training epochs were 100, we used a batch of 4 during training.

We compared our method with seven CNN-based methods, FCN [17], GCN [44], LinkNet [45], DeepLabv3 [32], U-Net [19], Attention U-Net [46], and M-Net [47]. Especially, FCN [17] combined with skip architecture for semantic segmentation. GCN [44] addressed classification and localization issues for semantic segmentation with a global convolutional network and boundary refinement block. LinkNet [45] was an encoder–decoder network with a small number of parameters. DeepLabv3 [32] used the ResNet as encoder and ASSP for capturing multi-scale context. U-Net [19] employed a symmetric encoder–decoder network with skip concatenation for biomedical image segmentation. Attention U-Net [46] improved the U-Net with an attention module. M-Net [47] adopted a U-shape CNN with the multi-label loss function for optic disc and cup segmentation.

We chose Dice loss as our segmentation loss function in Equation (10). Specifically, $C_{e}$ denoted the weight coefficient of each category, and e indicated each category of the segmentation task, e ∈ {0, 1}.${\;C}_{0}$ and $C_{1}$ were the weight coefficients of backgrounds and plaques, respectively. We set $C_{0}$ and $C_{1}$ as 0.5 in our experiment. $L_{e}$ and $S_{e}$ denoted the ground truth and the segmentation result of the category e. H and W represented the height and the width of the image used in our study, respectively.

(10) $$L_{\mathrm{Dice}}=\sum_{e=0}^{1}C_{e}\left\{1-\frac{2\times \sum_{m=1}^{H}\sum_{n=1}^{W}L_{e}\left(m,n\right)S_{e}\left(m,n\right)}{\sum_{m=1}^{H}\sum_{n=1}^{W}L_{e}\left(m,n\right)+\sum_{m=1}^{H}\sum_{n=1}^{W}S_{e}\left(m,n\right)}\right\}$$

## 4. Results

### 4.1. Compared with State-of-the-Art Methods

**Figure 7:**
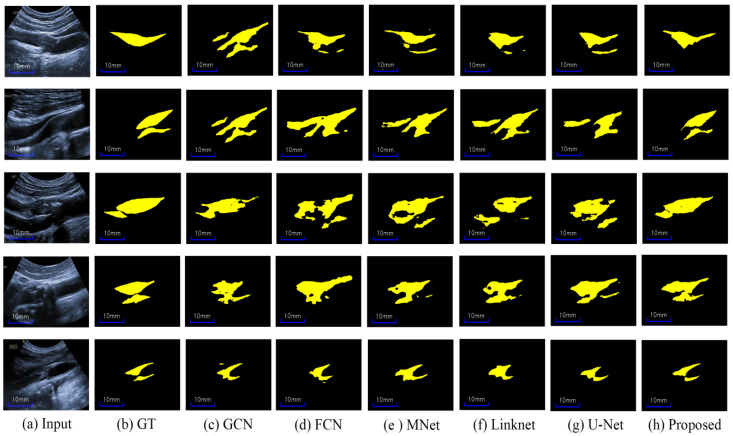
Results obtained by using different methods: (**a**) input images, (**b**) labels, (**c**) GCN, (**d**) FCN, (**e**) MNet, (**f**) Linknet, (**g**) U-Net, and (**h**) proposed method.

Herein, we first compare our proposed network with seven state-of-the-art CNN-based methods (described in the “Implementation Details” section) for a comprehensive assessment of our method for carotid ultrasound plaque segmentation. It is noteworthy that all methods are provided with the same data augmentation (GIA and CBV image augmentation), and other details are all the same for fairness in the training stages. Moreover, in Figure 3, the proposed HRU-Net can be easily replaced with other CNN-based methods in the plug-and-play structure. Table 2 lists the results of the different methods on the four metrics, and Figure 7 shows the segmentation predictions of representative plaques in ultrasound images by these methods. We also show three related works about plaque segmentation in the last three rows of Table 2.

**Figure 8:**
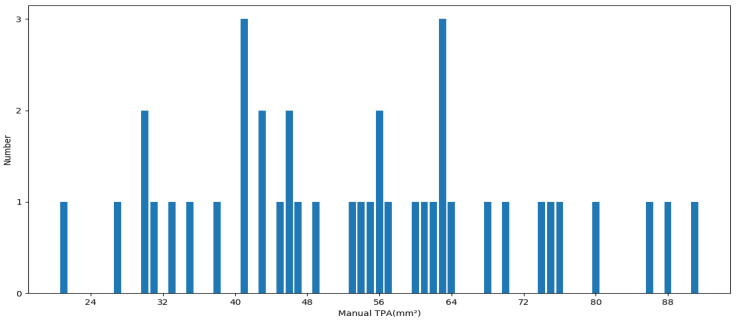
The total plaque areas’ distribution for 40 patients with severe stenotic plaques.

As can be seen from Table 2, our proposed method yields the best results on all metrics of the Dice (0.821 ± 0.053), IoU (0.701 ± 0.078), Acc (0.977 ± 0.008), and MHD (1.69 ± 1.46) compared to other methods. Moreover, all the metrics of the proposed method have the least standard variations. Noteworthy, the proposed method surpasses others by a large margin. Although the segmentation Dice of our method is not as high in the three related works, our plaque segmentation is more tough, as it contains various noises and heterogeneous plaques; moreover, the training images are less than the three related works. Furthermore, Figure 8 shows the area distribution of the 40 plaques; the proposed method obtains the ΔTPA with 2.81 mm^2^ ± 8.75 mm^2^ for the severe stenotic plaques.

To illustrate if there exist significant differences between two methods on the two popular metrics, Dice and MHD, we performed a paired *t*-test to compare the results of each baseline method with those of our method. The computed *p*-values are shown in Table 3. As can be found, all *p*-values are less than 0.05, suggesting that our results are significantly different from the results of other baseline methods; each improvement is statistically significant.

Figure 7 shows that our method outperforms the other methods in terms of visual results. In particular, the plaque-segmentation results by our method are closer to the labels with almost no background noise interference. Moreover, our method is more robust to the artifacts near the plaques and is capable of discriminating the plaques from serious speckle noise and low echo areas. Additionally, the proposed method could also discern ambiguous heterogeneous plaque boundaries and acoustic shadow regions for accurate plaque segmentation. In brief, our HRU-Net transfer-learning method retains better robustness to the large variability of carotid ultrasound-image qualities, further indicating the possibility of its wide clinical applicability.

### 4.2. Ablation Study

#### 4.2.1. Effect of Each Module

We performed an ablation study on the HRU-Net transfer-learning method and validated the performances of the introduced modules. It is worth noting that the general image augmentation (GIA) and RU-Net are used in each module.

The RU-Net with cropped blood vessel (CBV) image augmentation is abbreviated as RU-CBV. The RU-CBV with transfer learning is named RU-CBV-T. We named our proposed method without the CBV image augmentation HRU-Net-T. The proposed method also has an alternative name, HRU-CBV-T. Table 4 lists the ablation work results on the metrics of Dice, IoU, Acc, and MHD.

As shown in Table 4, the RU-Net had the lowest performance, and the segmentation accuracies were consistently improved relative to those of the RU-Net model by adding the introduced modules. Table 5 shows the paired *t*-test to compare each proposed module with the proposed method, and the improvements on the Dice metric are statistically significant. Subsequently, we explain the effects of the introduced modules in detail.

**Figure 9:**
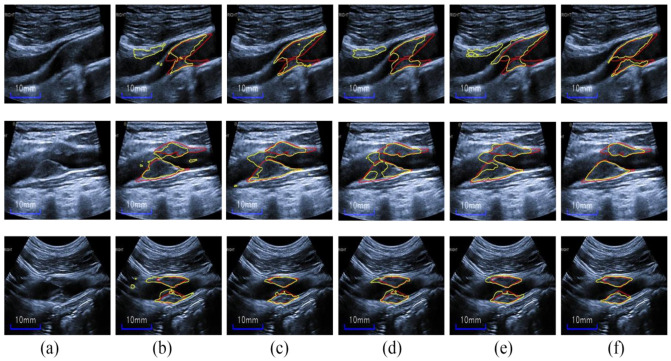
Transfer learning effect: (**a**) input images, (**b**) RU-CBV, (**c**) RU-CBV-T, (**d**) U-Net, (**e**) M-Net, and (**f**) HRU-CBV-T (proposed). The red and yellow outlines superimposed on the input images denote the labels and segmentation result contours.

**Transfer Learning:** As can be seen from the ablation work in Table 4, The RU-CBV-T including fine-tuning had an apparent increase of 0.053 on the Dice metric compared to RU-CBV without fine-tuning. As shown in the second and third columns of Figure 9, the RU-CBV-T including transfer learning acquired relatively more accurate and complete segmentation results than RU-CBV, the results of which are stained by background noise and artifacts.

In particular, the RU-CBV-T is also superior to the seven baseline methods on the metrics of Dice and MHD; it is observed that, in the fourth and fifth columns of Figure 9, RU-CBV-T acquires more accurate plaque segmentation results than U-Net and M-Net.

It is concluded that the knowledge from fine-grained classes of natural images could be transferred to the plaque segmentation in carotid ultrasound images, which have similar textures with artifacts or speckle noise.

**Figure 10:**
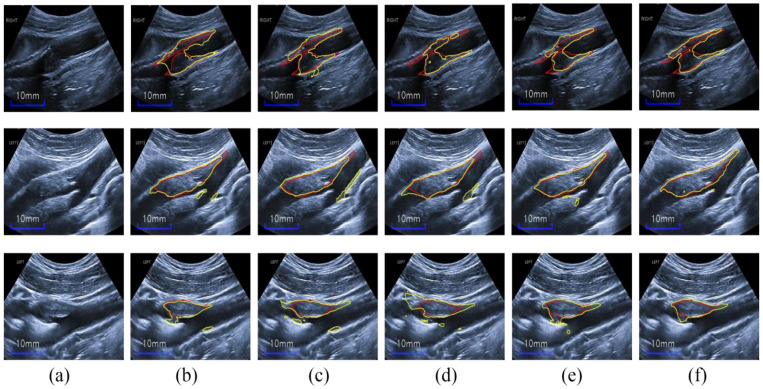
CBV effect: (**a**) input images, (**b**) M-Net, (**c**) U-Net, (**d**) Attention U-Net, (**e**) HRU-T, and (**f**) HRU-CBV-T (proposed). The red and yellow outlines superimposed on the input images denote the labels and segmentation-result contours.

**Cropped-Blood-Vessel Image Augmentation:** The HRU-CBV-T improves the Dice metric by 0.015 compared to HRU-T, and the CBV image augmentation could be served as an approach for fine segmentation of plaque boundaries, as shown in the fifth and sixth columns of Figure 10. The results of HRU-T mistake background and the speckle noise for plaques; meanwhile, the HRU-CBV-T makes a regularization pattern to the plaque segmentation result in the inner of the carotid artery and makes segmentation result more accurate.

**Figure 11:**
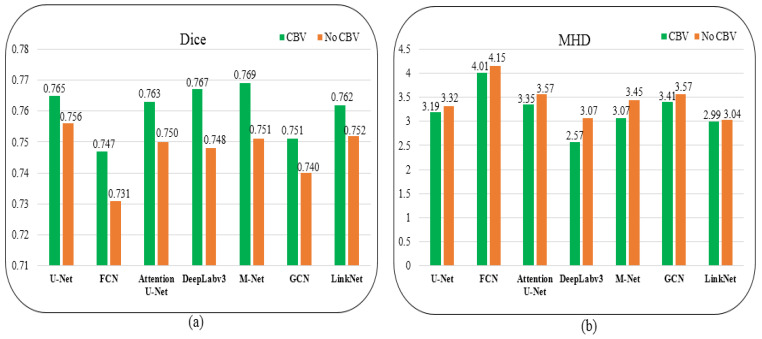
CBV effects on Dice metric (**a**) and MHD metric (**b**) of the seven cutting-edge methods.

We further evaluate the CBV effect on the other seven methods, and the results on Dice and MHD metrics are shown in Figure 11a,b, respectively. As can be observed from these two metrics, the seven methods (U-Net, FCN, Attention U-Net, DeepLabv3, M-Net, GCN, and LinkNet) with CBV obtained Dice values of 0.765, 0.747, 0.763, 0.767, 0.769, 0.751, and 0.762, respectively. Without the CBV during the training stage, the Dice values of these methods declined by 0.009, 0.016, 0.013, 0.019, 0.018, 0.011, and 0.01, respectively. In brief, the mean increase on Dice was 0.014 with the CBV in the training for these methods.

For the MHD metric, these methods without CBV produced MHD values of 3.32, 4.15, 3.57, 3.07, 3.45, 3.57, and 3.04, respectively. As a result of the CBV in the training, the MHD values increased by 0.13, 0.14, 0.22, 0.5, 0.48, 0.16, and 0.05, respectively, and the average promote value was 0.24. Hence, the CBV consistently improves the performance of all methods, and it is essential for better generalization during the training.

**Figure 12:**
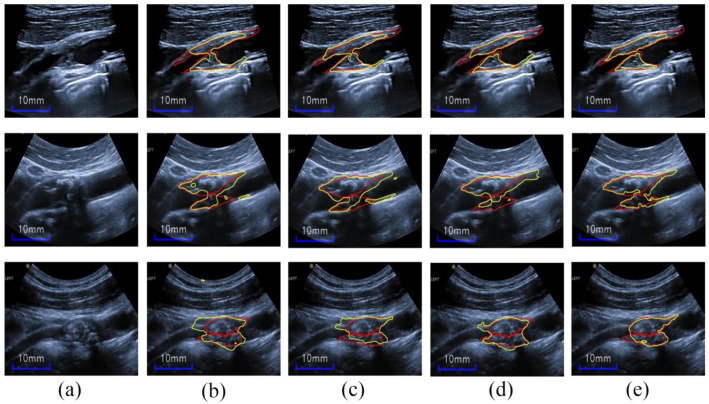
HAC effect: (**a**) input images, (**b**) M-Net, (**c**) U-Net, (**d**) RU-CBV-T, and (**e**) HRU-CBV-T (proposed). The red and yellow outlines superimposed on the input images denote the labels and segmentation result contours.

**Hybrid Atrous Convolutions:** The Dice value of the proposed method increases by 0.017 more than that of the RU-CBV-T, and the RU-CBV-T without the HAC can mistake speckle noise and blurry boundaries for plaques. In brief, the proposed method using the HAC module refines segmentation results for a better plaque-discrimination ability, as shown in the fourth and fifth columns of Figure 12.

The results indicate that the HAC modules (used on the three high-level layers) can acquire long-range dependency for plaques and suppress speckle noise and background information.

#### 4.2.2. Effect of the Number of HAC Modules

Furthermore, we evaluated the effect of the number of HAC modules for the proposed network. Table 6 shows the Dice values with different numbers of HACs. With the addition of the HAC module in the decoder from 1 to 6 (as shown in Figure 3), the segmentation Dice first increases from 0.812 to 0.821, and then it decreases from 0.821 to 0.808. As can be found, the Dice value reaches its peak value of 0.821 when the number of HACs is 3 (as shown in the last three deep layers of Figure 3).

It shows that the HAC module takes effect on the three high-level semantic layers, using latent distinguishable features about plaques, but obtains more irrelevant information on shallow layers (high-resolution features), resulting in the weakening of segmentation results in the decoding process.

### 4.3. Test Results on New Images

To further evaluate the performance of our method, we used 75 images that have never been seen in the training by the 10-fold trained models; moreover, these test images consist of 45 images with severe carotid stenosis and 30 images with moderate stenosis. Taking into account the characteristic of cross-validation, the pixel-level predictions were averaged over the softmax results for the trained 10-fold models. The test model of k-fold cross-validation can be expressed by Equation (11), where ${\mathrm{ModelPredict}}_{i}$ denotes the i-th trained model prediction result. In our paper, k is restricted to 10.

(11) $$\mathrm{test}\;\mathrm{model}=\frac{\sum_{i=1}^{k}{\mathrm{ModelPredict}}_{i}}{k}\;$$

**Figure 13:**
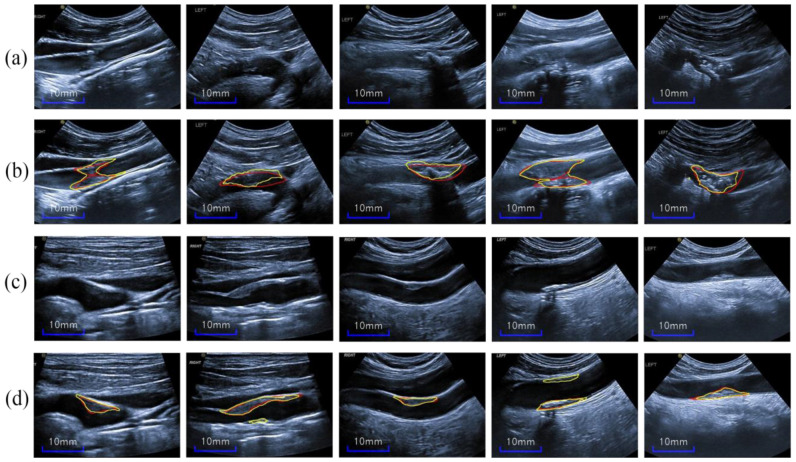
(**a**) and (**b**) show the test results on severe stenotic plaques, (**c**) and (**d**) display the test results on moderate stenotic plaques; the red and yellow outlines superimposed on the input images denote the labels and segmentation result contours.

**Figure 14:**
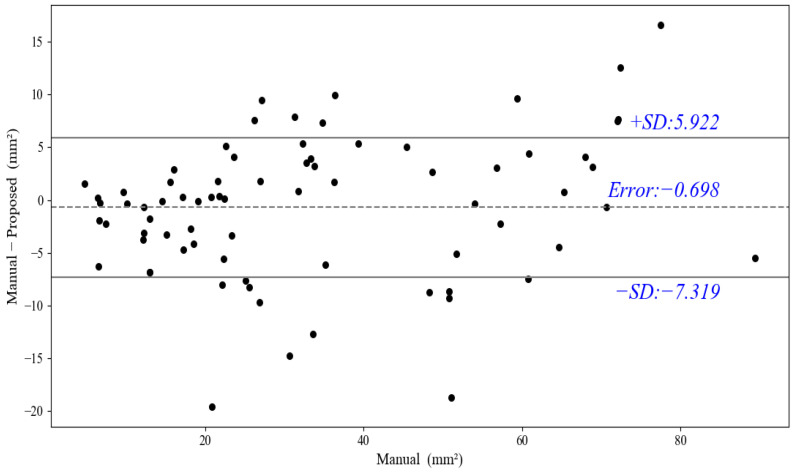
Plaque-area errors and standard deviations on new 75 test images.

Specifically, we obtained a Dice value of 0.805 ± 0.083, IOU of 0.682 ± 0.112, Acc of 0.985 ± 0.009, and MHD of 2.27 ± 2.92. Figure 13a,b shows the results of five severely stenotic images; our test model can accurately discern the unseen noisy plaques with confused boundaries, thus showing acceptable performance in terms of the four metrics. Additionally, as shown in Figure 13c,d, the images with moderate stenosis also show accurate and satisfactory segmentation performance; it is worth noting that these moderate stenotic plaques have never been seen in the training. Thus, this indicates that our test model shows fine generalization for moderate stenotic plaques; however, it may mistake IMC as plaques because of the similarity between the plaque and intima-media thickening region. Figure 14 further shows the plaque-area errors and standard deviations on the 75 test images; our method obtained the total plaque-area error −0.698 ± 6.62 mm^2^. The proposed method may be more useful for the plaques <70 mm^2^.

## 5. Discussion

Carotid vulnerable plaques are determinant factors for stroke risk because unstable plaques may rupture and result in emboli that travel into the brain and cause a stroke or transient ischemic attacks (TIAs). It is noteworthy that the segmentation of vulnerable plaques with severe stenosis can facilitate the plaque components’ identification and provide more information about the vulnerability of the plaques. However, severe stenotic plaque segmentation is still a difficult task for the reason of the blurry boundaries of plaques and the heterogeneities of inter-class and inner-class plaques. Furthermore, the limited images may result in a low segmentation performance for algorithms.

In this paper, we proposed an HRU-Net transfer-learning method, which is instantiated and improved from the common transfer learning, to segment carotid plaques by using limited ultrasound images. The proposed method comprises three sections: (1) The ResNet-50 encoder with a U-Net decoder structure is used for transfer learning. (2) An HACs (hybrid atrous convolutions) module is applied to obtain various long-range dependences of plaques for refined segmentation. (3) A cropped-artery-vessel data augmentation is adopted as a regularization mode for plaques’ segmentation only in the training phase; it decreases the overfitting for better generalization. The results show that our method obviously outperforms other CNN-based baseline methods and retains more accurate plaques boundaries.

The ablation experiment results show the effect of each proposed module. The RU-Net with transfer learning has an apparent increase on the Dice metric compared to the RU-Net without transfer learning; it even surpasses the second-best method, M-Net [47], by a large margin. Then we infer that the fine-tuning transfer learning from natural images could boost the segmentation performance with small samples, such as ultrasound medical images.

Furthermore, the CBV image augmentation refines the segmentation results of all methods used in this paper as a regularization mode, and the HAC module further enhances plaque contextual discrimination ability by obtaining multiple receptive fields on high-level layers of the network. The test results on new images show a fine generalization performance on not merely complex plaques with severe stenosis but also plaques with moderate stenosis.

**Figure 15:**
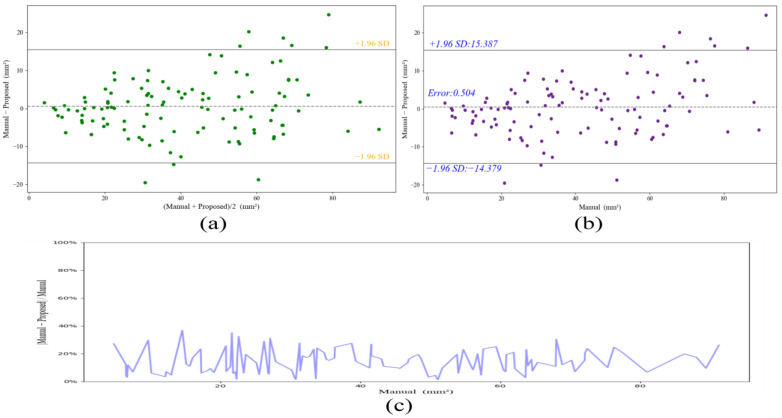
(**a**) Bland-Altman analysis of the segmentation results by our method on all 115 images. (**b**) Plaque-area errors and standard deviations on all 115 images. (**c**) Absolute percentage error versus plaque area among different plaques on all 115 images.

Additionally, we performed the Bland–Altman analysis on the segmentation results of all the 115 images. As can be seen from Figure 15a, our method can acquire relatively robust results compared to the ground truth. In particular, the proposed method can obtain better segmentation for plaques with a plaque area that is less 55 mm^2^, whereas the error may increase when the plaque areas are greater than 55 mm^2^. Furthermore, Figure 15b shows the plaque-area errors and standard deviations on all the images by our method; the proposed method obtains the total plaque-area error 0.5 ± 7.59 mm^2^, and the large standard deviations (SD) come from the severe stenotic plaques. The reason is that the heterogeneities of the stenotic plaques and severe speckle noise can make the plaque boundaries ambiguous and weaken the plaque segmentation inevitably. Moreover, Figure 15c shows the absolute percentage error versus plaque area by our method; the error is less than 40%, and the mean error is 14.2%, furthermore, the large error may exist in small plaques because these plaques may be disturbed by the intima-media complex or artifacts at the near wall of the carotid artery.

Moreover, Table 7 showed the comparisons of carotid plaque segmentation between our method and four CNN-based methods. In particular, for the Dice metric and plaque area error, Jain et al., (2021) first adopted hybrid deep-learning models [21], using 970 images to segment plaques; they obtained the mean Dice 88.98 ± 1.04 and best ΔPA 3.49 mm^2^ by using U-Net with CE-loss. Subsequently, they used the U-Net to segment plaques from two ethnic datasets [37] and validated the effects of Unseen AI; they obtained the Dice on the unseen AI pair one and two, and seen AI of 0.784 and 0.825, and 0.869, respectively. Moreover, Zhou et al., (2021) [20] utilized UNet++ ensemble to segment plaques on three small datasets and acquire the Dice 83.3–85.7 and ΔPA 0.73–6.75 mm^2^. Moreover, they also employed two U-Net segmenting plaques by using two different ground truths [36] and acquired the ΔTPA 0.05 ± 7.13 mm^2^ and 0.8 ± 8.7 mm^2^, respectively. Compared to the four studies, our HRU-Net obtained a Dice and ΔTPA of 0.811 and 0.5 ± 7.59 mm^2^, respectively; the ΔTPA of our method is comparable to the results of other papers, as shown in Table 7.

Although the segmentation Dice is not as high as these papers, our training images are only 40 which is less than these papers, our plaque segmentation task is more difficulty than theirs. Specifically, in the two papers [20,36] of Zhou et al., 2021, the patients with a carotid stenosis >70% were excluded, and the plaques in the images are clear without the noisy severely stenotic plaques. In the two studies [21,37] of Jain et al., 2021, only the far-wall plaques are segmented; however, the plaques on the far wall are ignored, and it is not reasonable in the clinic. In brief, we used the images with various plaque stenosis (10% < stenosis < 99%) both on the near and far walls of the carotid artery, as these are more useful in the clinic.

In summary, the proposed method obtained satisfactory segmentation results for the extremely heterogeneous plaques comparable to professional ultrasonic technicians and may serve as an alternative in clinical applications. Although some postprocessing methods such as snake [48], CRF [49], and continuous max flow [24] refine segmentation results by different theories, they may be not suitable for various noisy ultrasound images. Moreover, the abovementioned postprocessing methods easily fall into local optimum without sufficient prior knowledge.

Some limitations exist in our current work. Firstly, the 115 carotid ultrasound images are too few and insufficient to represent the actual distribution of plaques; plentiful ultrasound images should be acquired for deep-learning training and testing. Additionally, the segmentation result of the proposed method may be somehow affected by severe artifacts and speckle noise of ultrasound plaque images, as described above, and training the model by using more images with various stenotic plaques may mitigate these issues. More importantly, we will also try to use ultrasound video images to train the network in order to obtain better results, because video images support sequential plaque features, and the negative effects of artifacts and ambiguous boundaries may be inhibited by using the temporal frames.

It is worth noting that severe stenosis plaques have richer tissue-component information about the plaque instability [50] than moderate stenosis plaques. The components of severe stenosis plaques contain more tissues of hemorrhage, necrosis, lipids, fibers, and calcification. Furthermore, the identification and statistical analysis of these plaque components could reveal the complete development process of vulnerable plaques. Moreover, our methodology may help automatically segment plaques with severe stenosis in some studies [51,52,53] for discerning symptomatic and asymptomatic plaques with stenosis >70%. It is worth mentioning that the automated fine-grained identification of plaques components is the next priority for a comprehensive analysis of atherosclerosis as a supplementary means in the clinic.

## 6. Conclusions

In this paper, an HRU-Net transfer learning method for segmenting carotid plaques with severe stenosis in ultrasound images was proposed. A cropped-artery image augmentation method was utilized to restrain the segmented plaques in the artery only in training for the limited images. Obviously, the ResNet-50 encoder transfer learning with skip connections decoder structure enhances plaques’ recognition ability. In addition, HAC (hybrid atrous convolution) modules are capable of obtaining better context discrimination features on the three high-level semantic layers for precise segmentation. The results show that our method outperforms other baseline methods and is more robust to various noises, retaining more accurate plaque boundaries. Moreover, our automatic algorithm has a good generalization ability; it could be used for plaque identification before performing plaque texture analysis.

The proposed automatic plaque segmentation method could be useful for general clinicians to quantify the morphological features of plaques and to improve the objectivity and efficiency of plaque interpretation. We demonstrate that the automatic image segmentation system has the potential to serve as a supplementary method to identify and measure atherosclerosis plaques in carotid ultrasound images.

## Figures and Tables

**Table 1:** Encoder details of the ResNet-50.

Layer Name	Block	Output Size	Original Output Channels	Reduced Output Channels
CONV-Block	7 × 7, 64, stride 2	H/2 × W/2	64	32
Res1	$\left\{\begin{array}{c} 1\times 1,\;64\\ 3\times 3,\;64\\ 1\times 1,\;256 \end{array}\right\}\times 3$	H/4 × W/4	256	64
Res2	$\left\{\begin{array}{c} 1\times 1,\;128\\ 3\times 3,\;128\\ 1\times 1,\;512 \end{array}\right\}\times 4$	H/8 × W/8	512	96
Res3	$\left\{\begin{array}{c} 1\times 1,\;256\\ 3\times 3,\;256\\ 1\times 1,\;1024 \end{array}\right\}\times 6$	H/16 × W/16	1024	128
Res4	$\left\{\begin{array}{c} 1\times 1,\;512\\ 3\times 3,\;512\\ 1\times 1,\;2048 \end{array}\right\}\times 3$	H/32 × W/32	2048	128

**Table 2:** Comparison between different methods on our dataset and some results on relevant papers, where the numbers in bold indicate the best results. It is worth noting that CBV training augmentation is used in all the methods. NA: not applicable.

Methods	Dice	IoU	Acc	MHD
Proposed	**0.821 ± 0.053**	**0.701 ± 0.078**	**0.977 ± 0.008**	**1.69 ± 1.46**
U-Net [19]	0.765 ± 0.069	0.625 ± 0.091	0.969 ± 0.013	3.19 ± 2.62
FCN [17]	0.747 ± 0.073	0.601 ± 0.089	0.965 ± 0.015	4.01 ± 3.59
Attention U-Net [46]	0.763 ± 0.083	0.624 ± 0.105	0.968 ± 0.016	3.35 ± 3.52
DeepLabv3 [32]	0.767 ± 0.075	0.629 ± 0.098	0.969 ± 0.014	2.57 ± 1.79
M-Net [47]	0.769 ± 0.084	0.633 ± 0.108	0.968 ± 0.013	3.07 ± 2.70
GCN [44]	0.751 ± 0.095	0.610 ± 0.112	0.965 ± 0.021	3.41 ± 3.91
LinkNet [45]	0.762 ± 0.082	0.622 ± 0.105	0.967 ± 0.014	2.99 ± 2.65
Jain et al., 2021 [21]	0.889 ± 0.01	NA	NA	NA
Zhou et al., 2021 [20]	0.833–0.857	NA	NA	NA
Jain et al., 2021 [37]	0.784/0.825	NA	0.986/0.987	NA

**Table 3:** Paired *t*-test with Bonferroni correction of Dice and MHD metrics between the proposed method and others.

	U-Net	FCN	Attention U-Net	DeepLabv3	M-Net	GCN	LinkNet
Dice *p*-value	2.36 × 10^−6^	8.97 × 10^−7^	0.000107	3.31× 10^−5^	0.000403	0.00027	7.93 × 10^−5^
MHD *p*-value	0.0099	0.00246	0.00246	0.03335	0.014517	0.04184	0.03561

**Table 4:** Ablation work of the proposed method. The bold number denotes the best result.

GIA ^1^	CBV ^2^	T ^3^	HAC	Dice	IoU	Acc	MHD
✓				0.742 ± 0.069	0.594 ± 0.085	0.965 ± 0.011	3.53 ± 2.81
✓	✓			0.751 ± 0.088	0.609 ± 0.111	0.967 ± 0.013	3.13 ± 2.65
✓	✓	✓		0.804 ± 0.061	0.676 ± 0.086	0.974 ± 0.011	2.13 ± 1.53
✓		✓	✓	0.806 ± 0.056	0.679 ± 0.081	0.975 ± 0.010	2.06 ± 1.52
✓	✓	✓	✓	**0.821 ± 0.053**	**0.701 ± 0.078**	**0.977 ± 0.008**	**1.69 ± 1.46**

^1^ GIA denotes general image augmentation. ^2^ CBV denotes cropped blood vessel image augmentation. ^3^ T denotes transfer learning.

**Table 5:** Paired *t*-test with Bonferroni correction between the proposed and each ablated method.

Methods	*p*-Value of Dice	*p*-Value of MHD
RU-CBV vs. RU-CBV-T	0.002	0.047
RU-CBV vs. proposed	2.78 × 10^−5^	0.012
RU-CBV-T vs. proposed	0.042	0.374
HRU-T vs. proposed	0.035	0.12

**Table 6:** The effect with the increase of HAC module to the proposed network. The bold number denotes the best value.

HACs	1	2	3	4	5	6
Dice	0.812	0.815	**0.821**	0.818	0.814	0.808

**Table 7:** Plaque segmentation comparison between different studies.

Authors	Methods	Data	Metrics	Results
Jain et al., 2021 [21]	SDL/HDL models	97 patients/970 images	Jaccard, Dice, FoM, ΔPA	80.44 ± 1.59, 88.98 ± 1.04, 99.00 ± 1.10, best 3.49 mm^2^ using UNet with CE-loss
Zhou et al., 2021 [20]	UNet++ ensemble	144 patients/510 plaques	Dice, ΔPA, ICC, CoV	83.3–85.7, 0.73–6.75 mm^2^, 0.996, 6.98
Jain et al., 2021 [37]	UNet	165 Japanese patients/330 images 50 Hong Kong patients/300 images	mean accuracy, Dice, correlation-coefficient	Unseen AI pair one: 98.55, 0.784 and 0.80 Unseen AI pair two: 98.67, 0.825, and 0.87 Seen AI: 99.01, 0.869 and 0.92
Zhou et al., 2021 [36]	Two UNet	144 patients/510 plaques	ΔTPA, Pearson correlation	0.05 ± 7.13 mm^2^, 0.8 ± 8.7 mm^2^ 0.989, 0.987
Proposed	HRU-NET	90 patients/115 images	Dice, IOU, Acc, MHD, ΔTPA	0.811, 0.689, 0.982, 2.06, 0.5 ± 7.59 mm^2^

## Data Availability

The image data used to support the findings of this study are available from the corresponding author upon request.
